# Gleason score, surgical and distant metastasis are associated with cancer-specific survival and overall survival in middle aged high-risk prostate cancer: A population-based study

**DOI:** 10.3389/fpubh.2022.1028905

**Published:** 2022-10-18

**Authors:** Guangbiao Cao, Yan Li, Jinkui Wang, Xin Wu, Zhaoxia Zhang, Chenghao Zhanghuang, Keqiang Han

**Affiliations:** ^1^Department of Hepatobiliary Surgery, Songshan General Hospital, Chongqing, China; ^2^Department of Stomatology, Children's Hospital of Chongqing Medical University, Chongqing, China; ^3^Department of Urology, Children's Hospital of Chongqing Medical University, Chongqing, China

**Keywords:** prostate cancer, middle age, high-risk, SEER, nomogram

## Abstract

**Objective:**

According to statistics, patients with high-risk prostate cancer (PC) account for about 15% of prostate cancer diagnoses, and high-risk patients usually have a poor prognosis due to metastasis and recurrence and have a high mortality rate. Therefore, the accurate prediction of prognostic-related risk factors in middle-aged high-risk PC patients between 50 and 65 can help reduce patient mortality. We aimed to construct new nomograms for predicting cancer-specific survival (CSS) and Overall survival (OS) in middle-aged high-risk PC patients.

**Methods:**

Data for patients aged between 50 and 65 years old and diagnosed with high-risk PC were obtained from the Surveillance, Epidemiology, and End Results (SEER) database. Univariate and multivariate Cox regression models were used to identify independent risk factors for CSS and OS in patients. Nomograms predicting CSS and OS were developed based on multivariate Cox regression models. The concordance index (C-index), the area under the receiver operating characteristic curve (AUC), and the calibration curve are used to detect the accuracy and discrimination of the model. Decision curve analysis (DCA) is used to detect the potential clinical value of this model.

**Results:**

Between 2010 and 2018, 1,651 patients diagnosed with high-risk PC and aged 50–65 years were included. In this study, the training group (*n* = 1,146) and the validation group (*n* = 505) were randomly assigned in a ratio of 7:3. The results showed that M stage, Gleason (GS) and surgical mode were independent risk factors for CSS; marital status, T stage, M stage, surgical mode, and GS were independent risk factors for OS. The C-index for predicting CSS in the training and validation groups are 0.84 and 0.811, respectively; the C-index for predicting OS in the training and validation groups are 0.824 and 0.784, respectively. The AUC and the calibration curves also showed good accuracy and discrimination.

**Conclusions:**

We constructed new nomograms to predict CSS and OS in middle-aged high-risk PC patients. The prediction tools showed good accuracy and reliability, which can help clinicians and patients to make better clinical decisions.

## Background

Prostate cancer (PC) is the second most common malignancy in men after non-melanoma skin cancer (1). In 2022, there will be 2,608,490 new cases of prostate cancer in the US (2). Due to PSA detection technology's popularity, the PC detection rate is still increasing (3). In the past few decades, the incidence of prostate cancer in patients under 50 years old has increased by 5-fold (4), but PC is still at a high incidence over 50 years of age. The European Association of Urology (EAU) also recommends PSA testing for (5) in men over 50 years. According to statistics, patients with high-risk prostate cancer account for about 15% of (6) prostate cancer diagnoses. Moreover, high-risk patients usually have a poor prognosis due to metastasis and recurrence and have a high mortality (7). Therefore, it is crucial to find the influencing factors associated with overall survival (OS) and cancer-specific survival (CSS) in high-risk PC patients.

In the past, the traditional TNM staging system was mostly used for tumor evaluation, but it lacked many clinicopathological factors associated with cancer prognosis, such as surgical method, age, chemotherapy, etc. Although the D'Amico risk stratification system includes important indicators such as PSA and Gleason score (GS) for PC patients than the traditional TNM staging system, it still lacks key information such as patient marital status, race, age, etc. Risk stratification for PC patients currently relies on D'Amico: low risk (clinical stage T1-T2a, PSA<10 ng/mL and GS≤6), moderate risk (T2b or 10<PSA ≤20 ng/mL or GS 7), or high-risk (stage≥ T2c or PSA>20 ng/mL or GS≥8) (8). According to the literature, T stage, PSA and GS scores are all closely related to the prognosis of PC patients, so the prognosis of PC patients with different risks must be heterogeneous. High-risk PC patients have a poor prognosis due to their high PSA, GS and T stages, so it is particularly important to find factors related to the prognosis of high-risk PC patients.

The nomogram is essentially a graphical computational tool, it based on staging systems such as the American Joint Committee on Cancer (AJCC) and other key risk factors associated with prognosis to estimate the risk of disease (9). At present, more and more researchers develop nomograms based on SEER database to predict the survival and prognosis of various cancers. For example, Jiang et al. (10) developed the prognosis and nomogram to predict postoperative survival of duodenal adenocarcinoma, Zuo et al. (11) developed and constructed the survival nomogram for patients with stage IB non-small cell lung cancer, and Tang et al. (12) developed a novel nomogram to predict cancer-specific survival of patients initially diagnosed with metastatic oesophagal cancer. The incidence of PC is age-related, with more than 60% of patients over the age of 65 years old (13). However, it has been reported that 50 years is the most common cut-off age to distinguish between younger and older patients, with a median incidence of younger patients being 8.3%. Compared with the elderly patients in these consecutive cohorts, the younger group of patients below 50 consistently showed significantly more favorable clinicopathological features and a better oncological prognosis (14). Zheng et al. (15) suggested that age 50 may be a practical and meaningful cut-off value for when studying the effect of age on PC progression and considering treatment options. Unfortunately, to the best of our knowledge, no investigator have constructed nomograms for middle-aged high-risk PC patients between 50 and 65 years. Based on this, we constructed nomograms that can predict CSS and OS based on data from the SEER database, which underwent internal cross-validation to provide better guidance for clinicians.

## Methods

### Data source and extraction

Data on patients diagnosed with high-risk prostate cancer from the periods between 2010 and 2018, aged between 50 and 65 years, were downloaded from the SEER database. The SEER database contains data from 18 cancer medical centers and 30% of the population. The data in the SEER database are publicly available, and the patient information is hidden, so neither ethical approval nor informed consent from the patients is required. We followed the guidelines published in the SEER database for this study.

**Figure 1:**
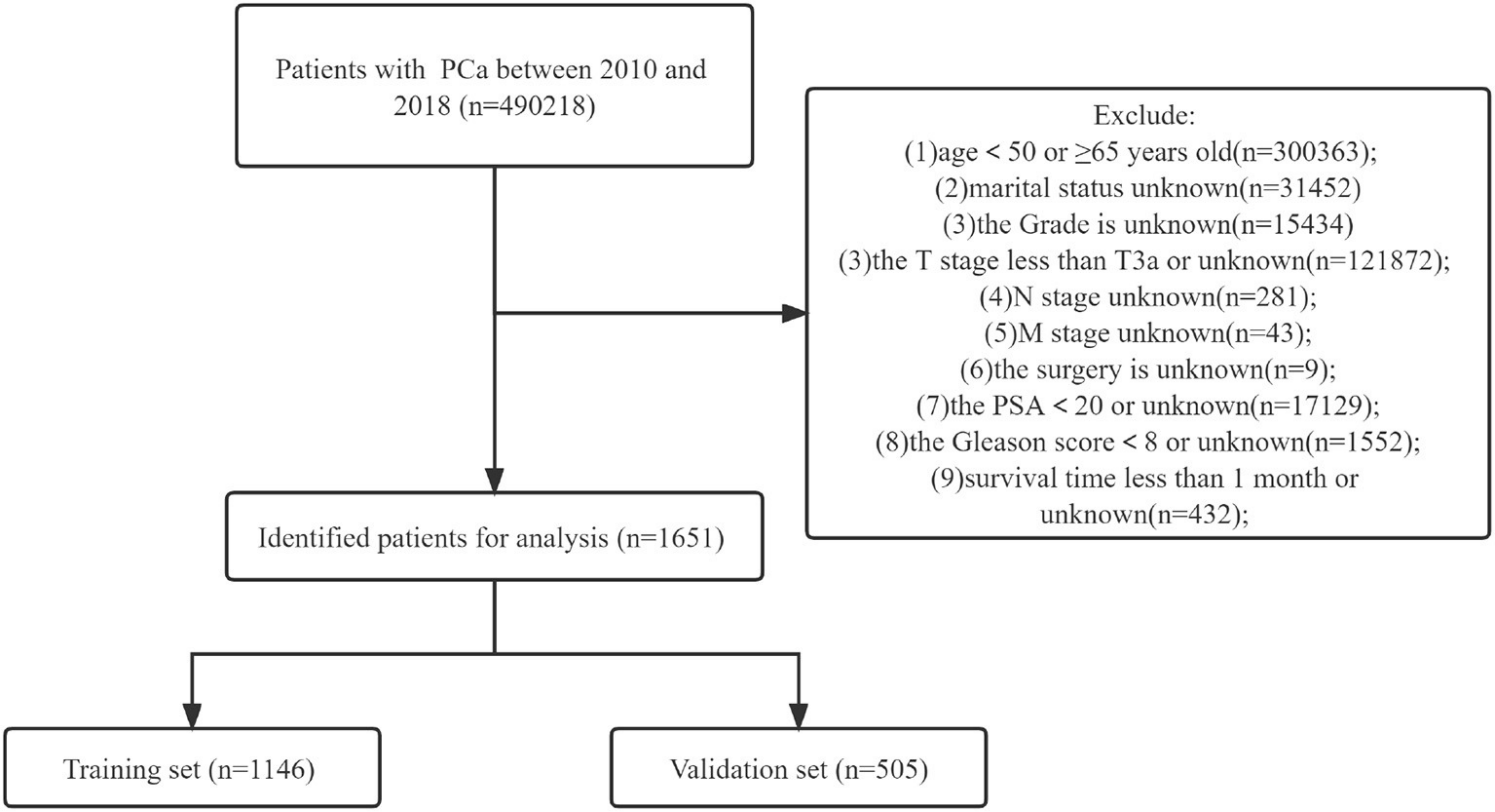
Flowchart for inclusion and exclusion of middle aged high-risk prostate cancer.

The SEER database includes many variables, such as race, age, marital status, surgical method, TNM stage, radiation therapy, tumor grade, chemotherapy, GS, and PSA. In addition, patient survival status, survival time, and cause of death were also obtained from the SEER database. The race of patients were classified as African American, Caucasian, and other. Marital status included married, single, divorced, and widowed. Surgical methods mainly include radical prostatectomy and local tumor resection. The inclusion criteria for this study were: (1) patients aged between 50 and 65 years; (2) a pathological diagnosis of prostate cancer; (3) PC patients with high-risk risk stratification by D'Amico. Exclusion criteria: (1) patients <50 or older than 65 years; (2) unknown marital status; (3) unknown tumor grade; (4) T stage below T3a or T stage unknown; (5) N stage unknown; (6) M stage unknown; (7) unknown surgical method; (8) PSA < 20 ng/ml or unknown; (9) GS score <8 or unknown; (10) survival time <1 month or unknown survival time. A flowchart of patient inclusion and exclusion is shown in Figure 1.

### Development and validation of the nomograms

In this study, a total of 1,651 middle-aged high-risk PC patients were included. We set a random number and randomly divided all patients into training and validation groups in a ratio of 7:3. Independent risk factors for patients in the training set were analyzed using univariate and multivariate Cox proportional regression models. Univariate Cox regression analysis was performed on all variables to screen for prognostic factors. Multivariate Cox stepwise backward regression analysis was then used to identify independent risk factors. Nomograms were constructed based on the results of the multivariable Cox regression analysis and were used to predict the CSS and OS at 3-, 5-, and 8- years in middle-aged high-risk PC patients. In addition, we used a calibration curve of 1,000 bootstrapped samples to validate the prediction accuracy of the nomogram at 3, 5, and 8 years. Finally, we used the area under the receiver operating characteristic curve (AUC) test and the concordance index (C-index) to test the accuracy and discrimination of the model.

### Clinical application

The potential clinical value of nomograms were assessed using decision analysis curves (DCA). In addition, the risk scores for each patient was calculated. Based on the receiver operating characteristic (ROC) curve, we used the Youden index to choose the best cutoff value. Based on this cutoff value, patients were divided into high-risk and low-risk groups. Using log-rank test and Kaplan-Meier (K-M) curve, we tested the differences in survival between high-risk and low-risk patients. We analyzed the CSS and OS of patients between different surgery, GS, and metastasis. In addition, we analyzed differences in OS for T stage and marital status between high- and low-risk groups.

### Statistical analysis

Continuous variables are described using mean ± standard deviation. Comparisons between groups were performed using the chi-square or non-parametric *U*-test. Frequency (%) was used to describe categorical variables and chi-square test was used to compare differences between groups. Cox regression model was used to analyze the prognostic factors of patients, and survival differences were analyzed by K-M curve and log-rank test. R software version 4.1.0 and SPSS 26.0 were used for statistical analysis. R packages include “survival,” “ggdca,” “dynnom” and “rms.” A *p*-value < 0.05 were considered statistically significant.

## Results

### Clinical features

In total, 1,651 patients with patients with PC were included in this study by the inclusion exclusion criteria. These patients were all aged between 50 and 65 years old, and were diagnosed as high-risk. The training group consisted of 1,146 patients and the validation group consisted of 505 patients. The mean age of all patients was 58.7 ± 3.94 years, with 71.3% Caucasian and 61.0% married. The tumor grade was dominated by grade III (99.7%). The T stages were T3a (28.0%), T3b (43.8%), and T4 (23.2%). The N stages were N0 (53.1%) and N1 (46.9%). The majority of the patients were mainly M0 (74.2%). Patients with radical prostatectomy were 51.7%, and 43.4% without surgery. Patients who did not receive chemotherapy accounted for 99%. While 37.8% of patients did not receive radiotherapy, the majority of patients received radiotherapy, including beam radiation (0.42%), radioactive implants or isotopes (1.76%), and combined radiotherapy (60.0%). The GS score of eight points was 39.6%, GS 9 points 51.5% and GS 10 points 8.90%. There were 53.8% of patients with PSA ≥20 ng/ml and <50 ng/ml, 20.5% with PSA≥50 ng/ml and <98 ng/ml, and 25.6% with PSA≥98 ng/ml. Table 1 shows the clinical characteristics of the two groups of patients, and the results showed no significant statistical bias.

**Table 1:** Clinicopathological characteristics of patients with high-risk PC in middle age.

	**All**	**Training cohort**	**Validation cohort**	
	***N* = 1,651**	***N* = 1,146**	***N* = 505**	***p***
Age	58.7 (3.94)	58.7 (3.98)	58.9 (3.84)	0.164
Race:				0.761
Caucasian	1,177 (71.3%)	821 (71.6%)	356 (70.5%)	
African American	339 (20.5%)	235 (20.5%)	104 (20.6%)	
Other	135 (8.18%)	90 (7.85%)	45 (8.91%)	
Marital:				0.508
Married	1,007 (61.0%)	694 (60.6%)	313 (62.0%)	
Single	392 (23.7%)	281 (24.5%)	111 (22.0%)	
Divorced or widowed	252 (15.3%)	171 (14.9%)	81 (16.0%)	
Grade:				0.367
II	1 (0.06%)	0 (0.00%)	1 (0.20%)	
III	1,646 (99.7%)	1,143 (99.7%)	503 (99.6%)	
IV	4 (0.24%)	3 (0.26%)	1 (0.20%)	
T:				0.082
T3a	463 (28.0%)	330 (28.8%)	133 (26.3%)	
T3b	723 (43.8%)	512 (44.7%)	211 (41.8%)	
T4	465 (28.2%)	304 (26.5%)	161 (31.9%)	
N:				0.793
N0	876 (53.1%)	611 (53.3%)	265 (52.5%)	
N1	775 (46.9%)	535 (46.7%)	240 (47.5%)	
M:				0.137
M0	1,225 (74.2%)	863 (75.3%)	362 (71.7%)	
M1	426 (25.8%)	283 (24.7%)	143 (28.3%)	
Surgery:				0.100
No	716 (43.4%)	478 (41.7%)	238 (47.1%)	
Local tumor excision	82 (4.97%)	56 (4.89%)	26 (5.15%)	
Radical prostatectomy	853 (51.7%)	612 (53.4%)	241 (47.7%)	
Chemotherapy:				0.885
No	133,822 (99.0%)	40,015 (99.0%)	93,807 (99.0%)	
Yes	1,361 (1.00%)	404 (1.00%)	957 (1.01%)	
Radiation:				0.663
No	624 (37.8%)	427 (37.3%)	197 (39.0%)	
Beam radiation	7 (0.42%)	4 (0.35%)	3 (0.59%)	
Radioactive implants or isotopes	29 (1.76%)	19 (1.66%)	10 (1.98%)	
Combination	991 (60.0%)	696 (60.7%)	295 (58.4%)	
Gleason:				0.235
8	653 (39.6%)	447 (39.0%)	206 (40.8%)	
9	851 (51.5%)	588 (51.3%)	263 (52.1%)	
10	147 (8.90%)	111 (9.69%)	36 (7.13%)	
PSA:				0.535
20 ≤ PSA < 50	889 (53.8%)	626 (54.6%)	263 (52.1%)	
50 ≤ PSA < 98	339 (20.5%)	235 (20.5%)	104 (20.6%)	
PSA≥98	423 (25.6%)	285 (24.9%)	138 (27.3%)	
CSS:				0.154
Dead	292 (17.7%)	192 (16.8%)	100 (19.8%)	
Alive	1,359 (82.3%)	954 (83.2%)	405 (80.2%)	
Survival months	39.1 (25.6)	39.3 (25.7)	38.6 (25.4)	0.603
OS:				0.193
Dead	361 (21.9%)	240 (20.9%)	121 (24.0%)	
Alive	1,290 (78.1%)	906 (79.1%)	384 (76.0%)	

### Cox regression analysis

We first used a univariate Cox regression model to analyze the influencing factors of the training group related to CSS and OS. The results showed that surgery, TNM stage, chemotherapy, marital status, combined radiotherapy, GS, and PSA were prognostic factors affecting patients' CSS. While race, marital status, surgery, chemotherapy, TNM stage, combined radiotherapy, GS, and PSA were the influencing factors patients' OS. Then, independent risk factors associated with patients' CSS and OS were identified using multivariate Cox regression analysis. The results showed that M stage, surgery, and GS were independent risk factors for CSS. Whereas, T stage, marital status, M stage, surgery, and GS were independent risk factors for OS. The results are shown in Tables 2, 3.

**Table 2:** Univariate and multivariate analyses of CSS in training cohort.

	**Univariate**			**Multivariate**		
	**HR**	**95% CI**	***P***	**HR**	**95% CI**	***P***
Age	0.99	0.95–1.02	0.526			
Race:						
Caucasian						
African American	1.33	0.95–1.86	0.101			
Other	1.04	0.61–1.78	0.876			
Marital:						
Married						
Single	1.64	1.18–2.28	0.003			
Divorced or widowed	1.88	1.29–2.74	0.001			
T:						
T3a						
T3b	1.21	0.78–1.87	0.385			
T4	3.91	2.61–5.84	< 0.001			
N:						
N0						
N1	1.81	1.35–2.41	< 0.001			
M:						
M0						
M1	9.07	6.7–12.28	< 0.001	4.154	2.911–5.929	< 0.001
Surgery:						
No						
Local tumor excision	2.14	1.42–3.22	< 0.001	1.41	0.925–2.15	0.111
Radical prostatectomy	0.14	0.09–0.2	< 0.001	0.32	0.201–0.508	< 0.001
Chemotherapy:						
No						
Yes	2.57	1.79–3.69	< 0.001			
Radiation:						
No						
Beam radiation	0	0-Inf	0.994			
Radioactive implants or isotopes	1.04	0.32–3.32	0.951			
Combination	1.76	1.28–2.41	< 0.001			
Gleason:						
8						
9	2.34	1.63–3.36	< 0.001	1.715	1.19–2.471	0.004
10	5.8	3.74–8.98	< 0.001	2.664	1.693–4.194	< 0.001
PSA:						
20 ≤ PSA < 50						
50 ≤ PSA < 98	1.6	1.05–2.43	0.027			
PSA≥98	4.85	3.51–6.71	< 0.001			

**Table 3:** Univariate and multivariate analyses of OS in training cohort.

	**Univariate**			**Multivariate**		
	**HR**	**95% CI**	***P***	**HR**	**95% CI**	***P***
Age	1	0.97–1.04	0.876			
Race:						
Caucasian						
African American	1.42	1.06–1.91	0.019			
Other	0.89	0.53–1.49	0.668			
Marital:						
Married						
Single	1.78	1.33–2.38	< 0.001	1.254	0.983–1.599	0.068
Divorced or widowed	1.91	1.36–2.68	< 0.001	1.838	1.402–2.411	< 0.001
T:						
T3a						
T3b	1.13	0.77–1.65	0.536	1.224	0.89–1.684	0.214
T4	3.51	2.47–4.99	< 0.001	1.702	1.246–2.324	0.001
N:						
N0						
N1	1.58	1.23–2.05	< 0.001			
M:						
M0						
M1	7.05	5.42–9.17	< 0.001	2.984	2.305–3.865	< 0.001
Surgery:						
No						
Local tumor excision	2.01	1.37–2.93	< 0.001	1.27	0.916–1.76	0.151
Radical prostatectomy	0.17	0.12–0.24	< 0.001	0.377	0.274–0.52	< 0.001
Chemotherapy:						
No						
Yes	2.14	1.52–3.01	< 0.001			
Radiation:						
No						
Beam radiation	0	0-Inf	0.993			
Radioactive implants or isotopes	0.82	0.26–2.62	0.743			
Combination	1.75	1.32–2.32	< 0.001			
Gleason:						
8						
9	2.05	1.5–2.81	< 0.001	1.404	1.096–1.8	0.007
10	4.7	3.18–6.94	< 0.001	1.962	1.412–2.726	< 0.001
PSA:						
20 ≤ PSA < 50						
50 ≤ PSA < 98	1.47	1.02–2.13	0.041			
PSA≥98	4.38	3.29–5.83	0			

### Development and validation of the nomograms

**Figure 2:**
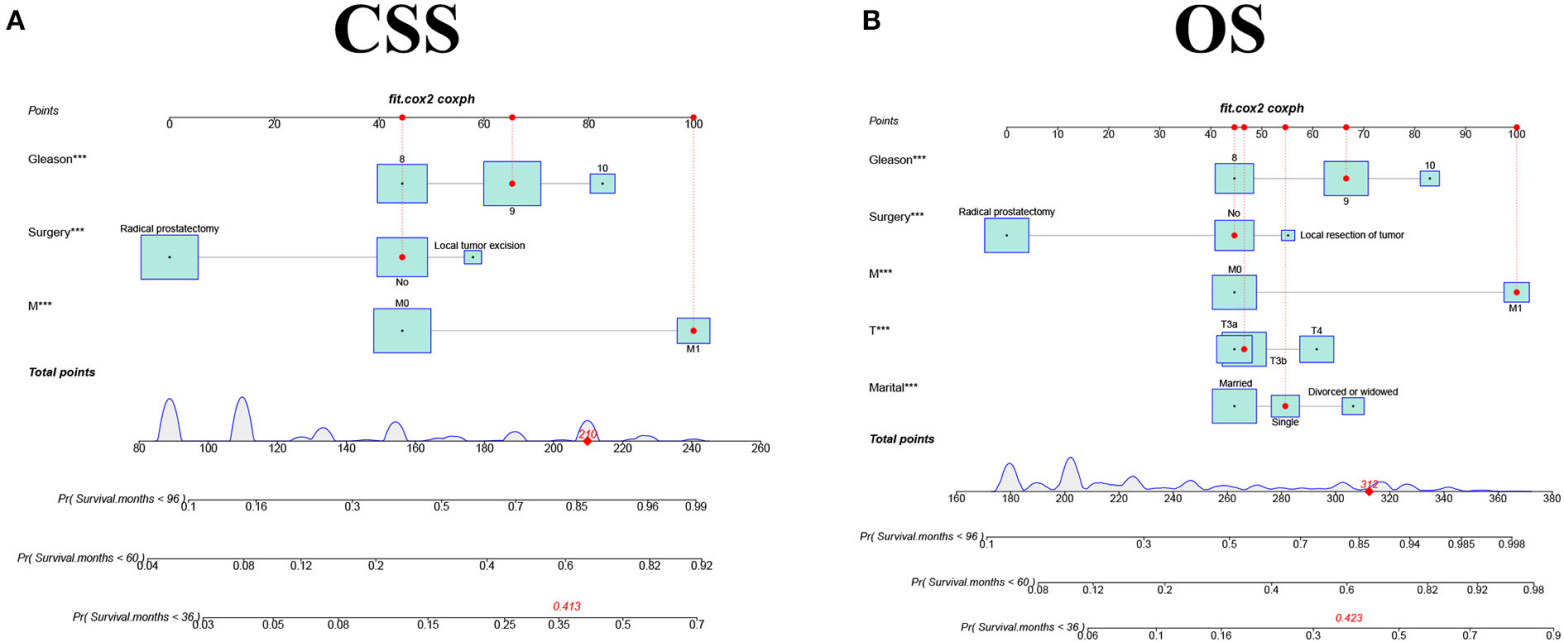
The nomograms for predicting 3-, 5-, and 8-year CSS and OS in middle aged high-risk prostate cancer. **(A)** The nomogram for predicting CSS. **(B)** The nomogram for predicting OS.

We constructed two nomograms based on a multivariate Cox regression analysis model to predict the 3-, 5-, and 8-year CSS (and OS) in middle-aged high-risk prostate cancer patients (Figure 2). As can be seen from the figure, M stage, surgical mode, and GS are independent risk factors for patients' CSS; marital status, T stage, M stage, surgical mode, and GS are independent risk factors affecting patients' OS.

**Figure 3:**
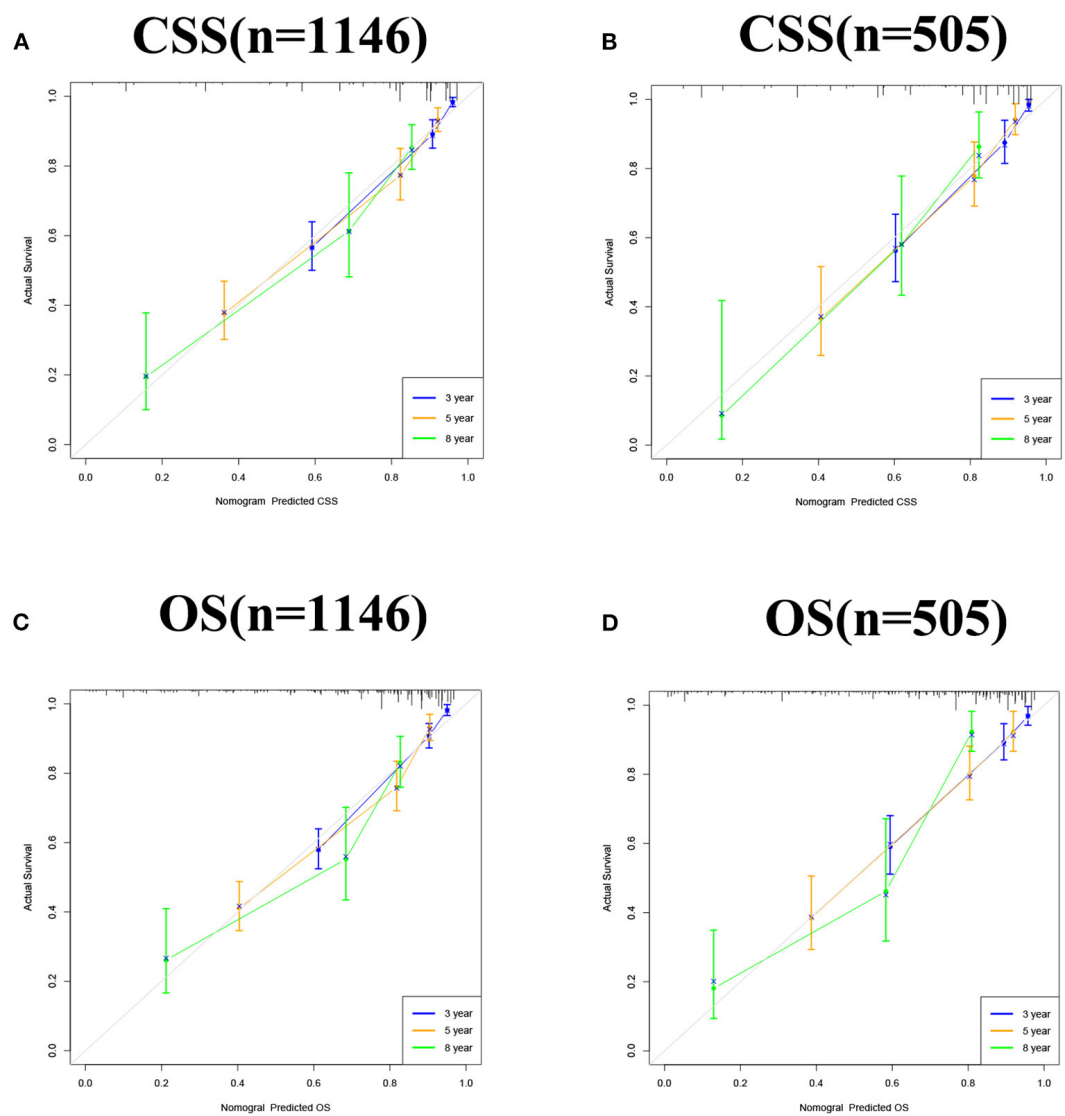
Calibration curve of the nomograms for predicting 3-, 5-, and 8-year CSS and OS in middle aged high-risk prostate cancer. **(A)** Calibration curve of the nomograms for predicting 3-, 5-, and 8-year CSS in the training set. **(B)** Calibration curve of the nomograms for predicting 3-, 5-, and 8-year CSS in the validation set. **(C)** Calibration curve of the nomograms for predicting 3-, 5-, and 8-year OS in the training set. **(D)** Calibration curve of the nomograms for predicting 3-, 5-, and 8-year OS in the validation set. The horizontal axis is the predicted value in the nomogram, and the vertical axis is the observed value.

**Figure 4:**
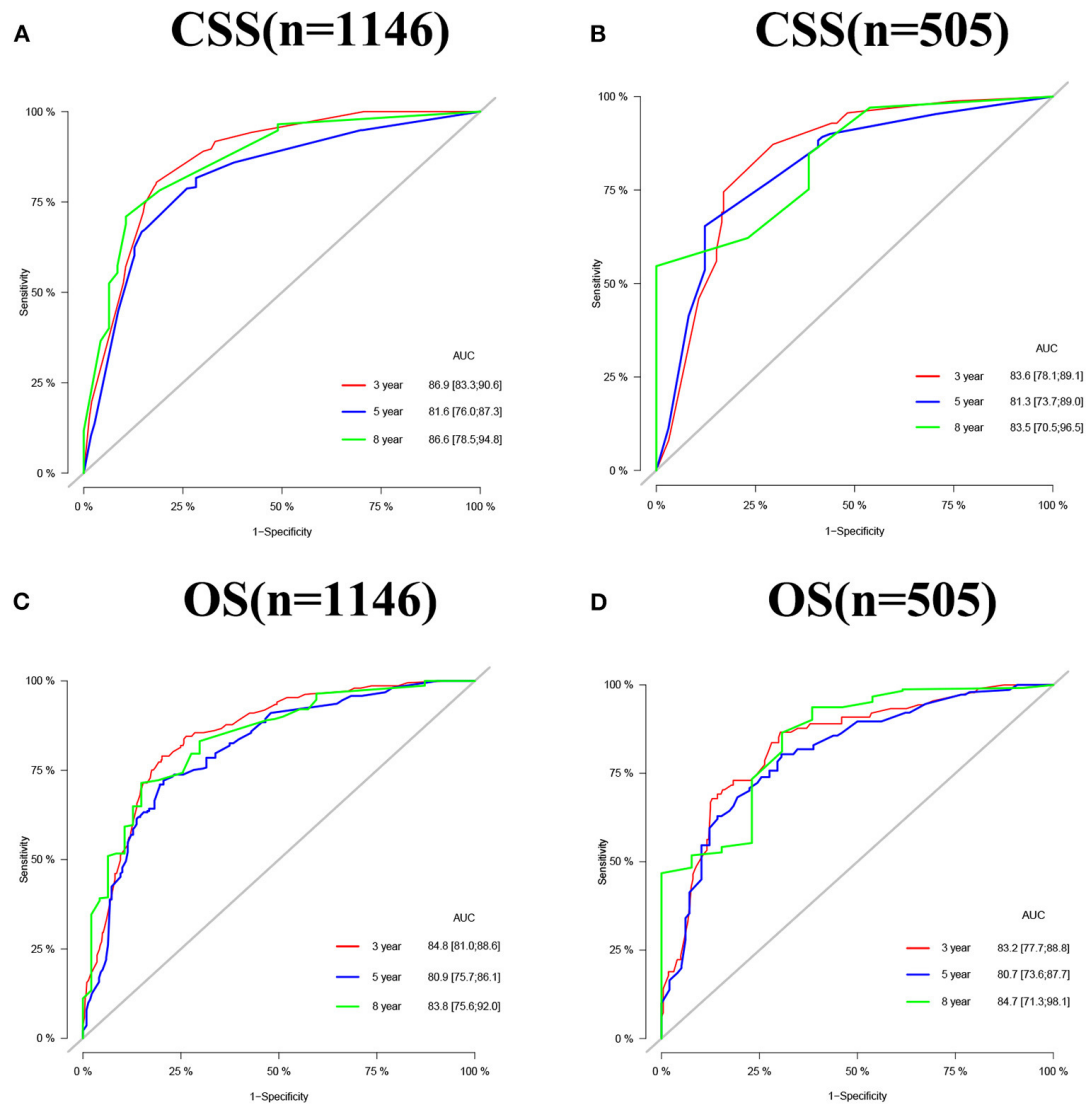
AUC for predicting 3-, 5-, and 8-year CSS **(A,B)** and OS **(C,D)** in middle aged high-risk prostate cancer.

The accuracy and discriminant of nomograms were evaluated by internal cross-validation. C-indexes for training and validation groups for predicting CSS are 0.84 (95% CI: 0.816–0.864) and 0.811 (95% CI: 0.772–0.85), respectively. C-indexes for training and validation groups for predicting OS are 0.824 (95% CI: 0.8–0.848) and 0.784 (95% CI: 0.741–0.827), respectively. It shows that the prediction models have good recognition ability. The results of calibration curves showed that the predicted values of the nomograms were highly consistent with the actually observed values (Figure 3), indicating that the nomograms have good accuracy. In the training group, AUC of the nomogram for CSS are 86.9, 81.6, and 86.6 at 3-, 5-, and 8-year. In the validation group, AUC of the nomogram for CSS are 83.6, 81.3, and 83.8. In the training group, AUC of the nomogram for OS are 84.8, 80.9, and 83.8. In the validation group, AUC of the nomogram for OS are 83.2, 80.7, and 84.7 (Figure 4).

### Clinical application of the nomograms

**Figure 5:**
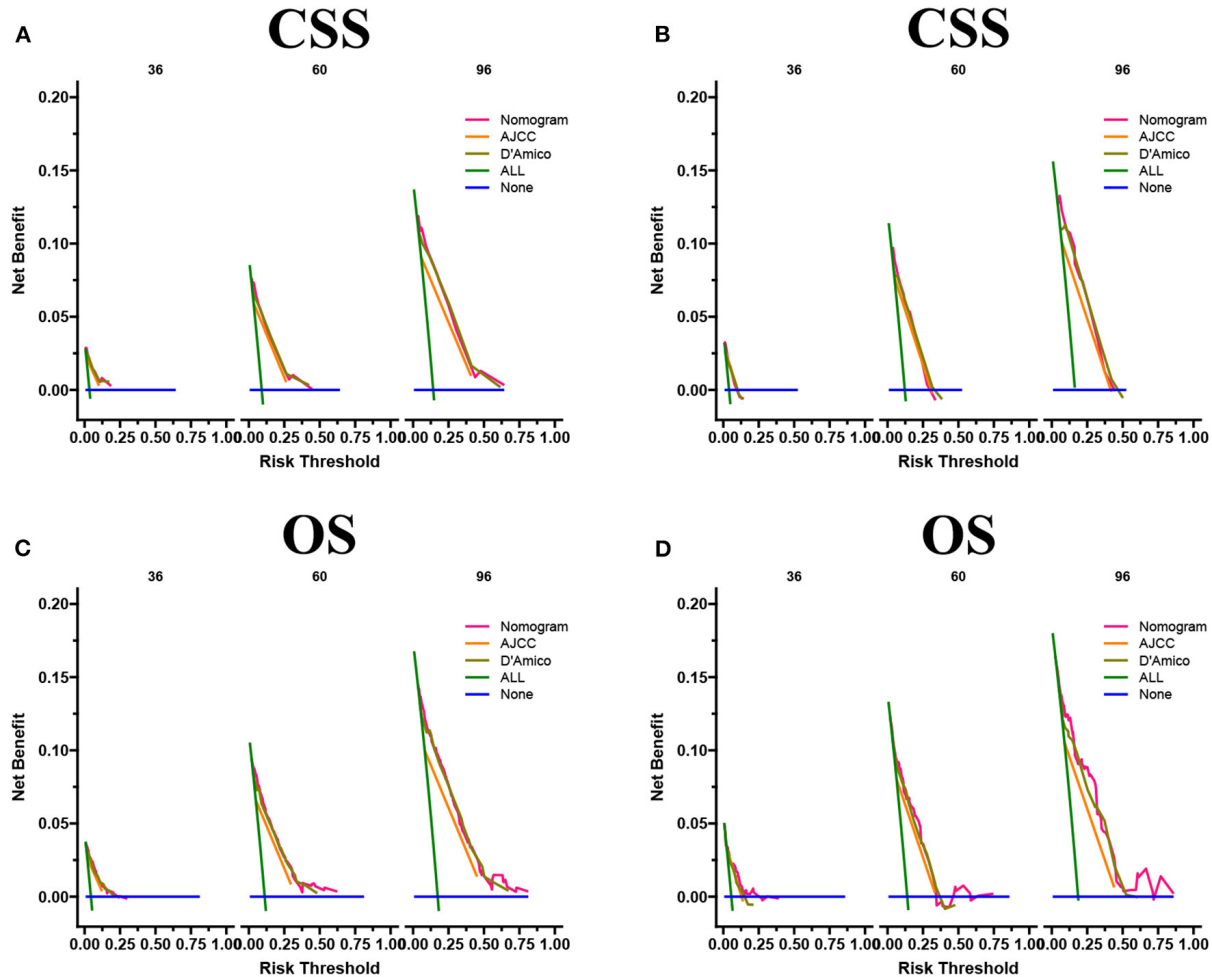
DCA of the nomograms for predicting CSS **(A,B)** and OS **(C,D)** of the training set and validation set.

**Figure 6:**
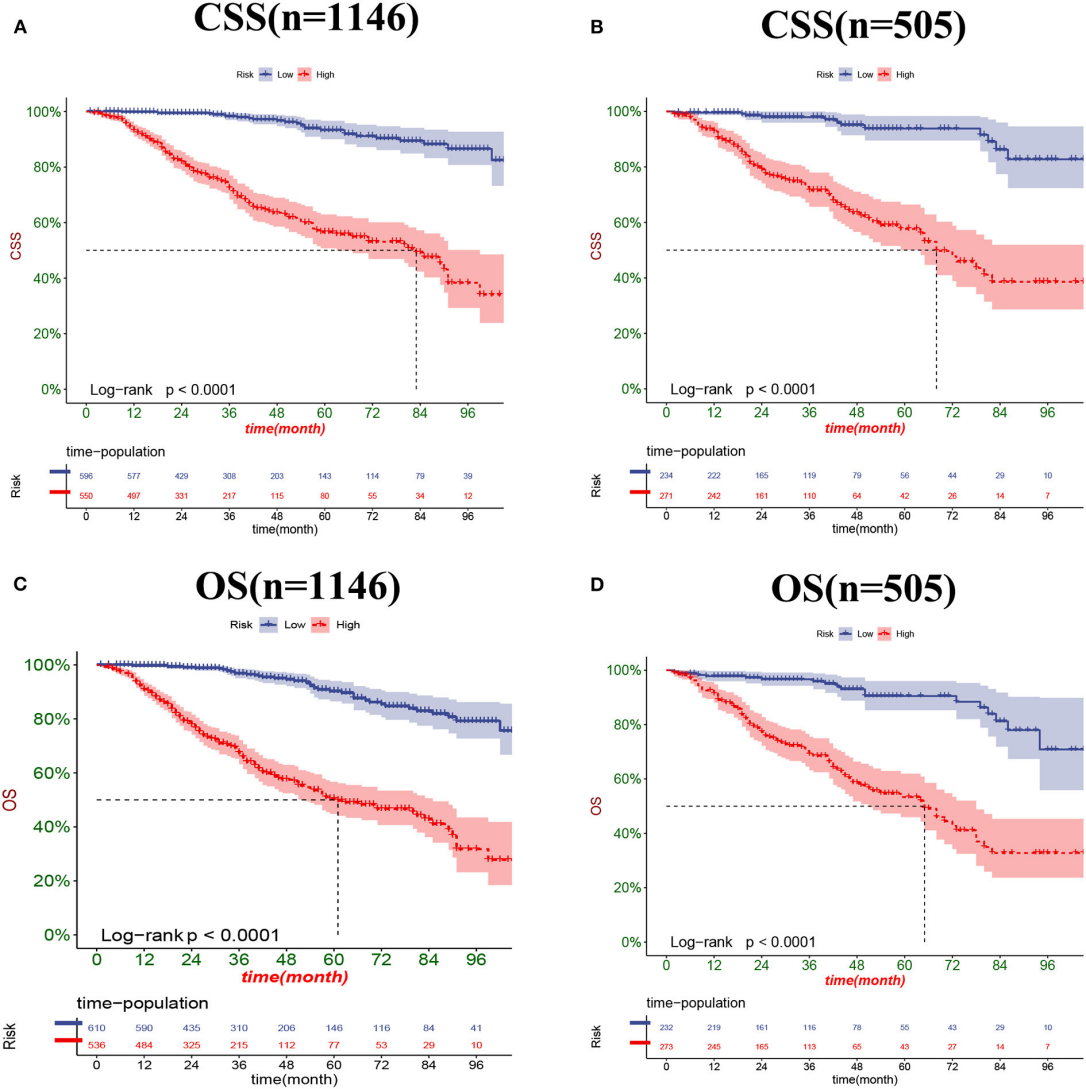
Kaplan-Meier curves for predicting CSS **(A,B)** and OS **(C,D)** of patients in the low-risk and high-risk groups.

**Figure 7:**
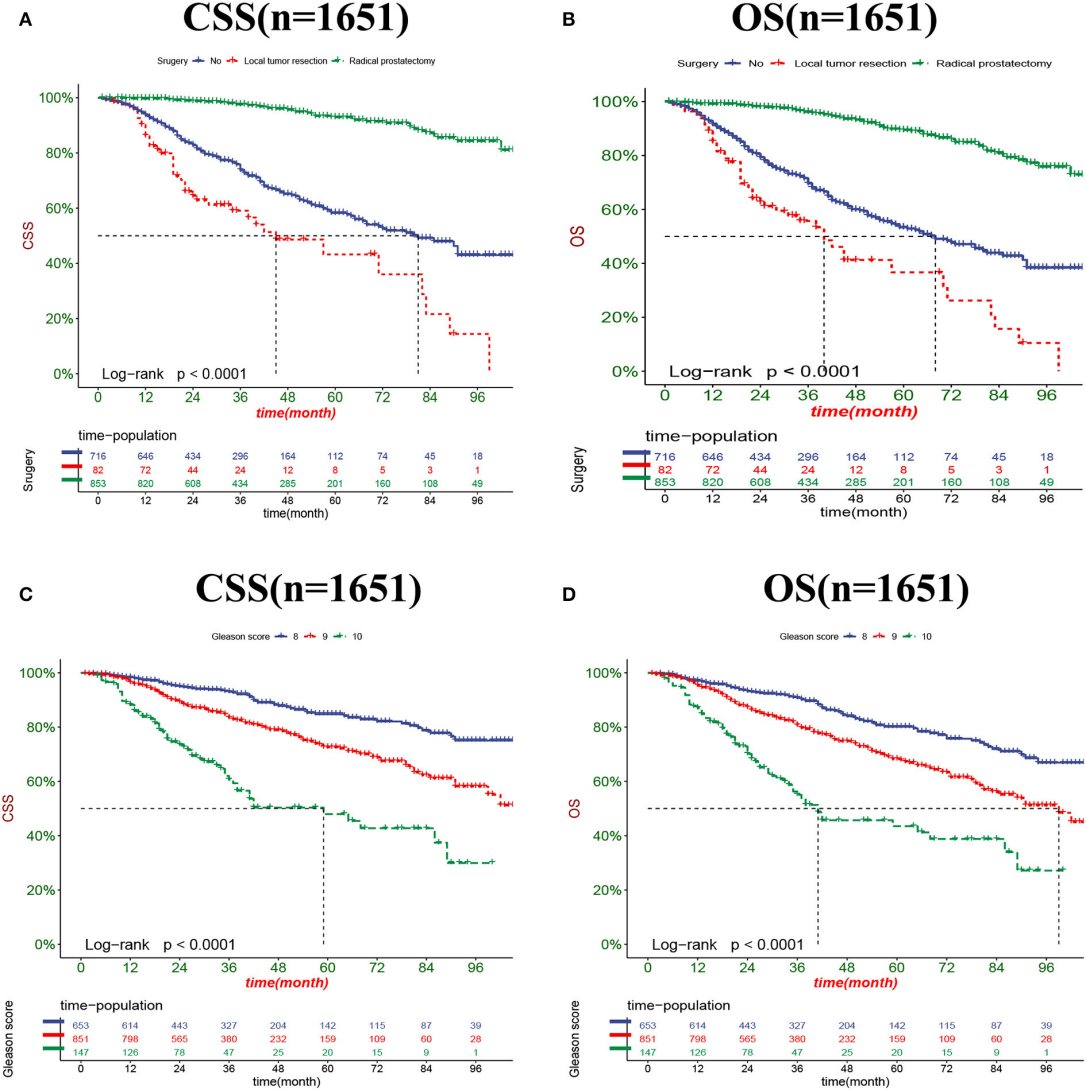
Kaplan-Meier curves for predicting CSS and OS in patients with different surgery **(A,B)** and GS **(C,D)**.

**Figure 8:**
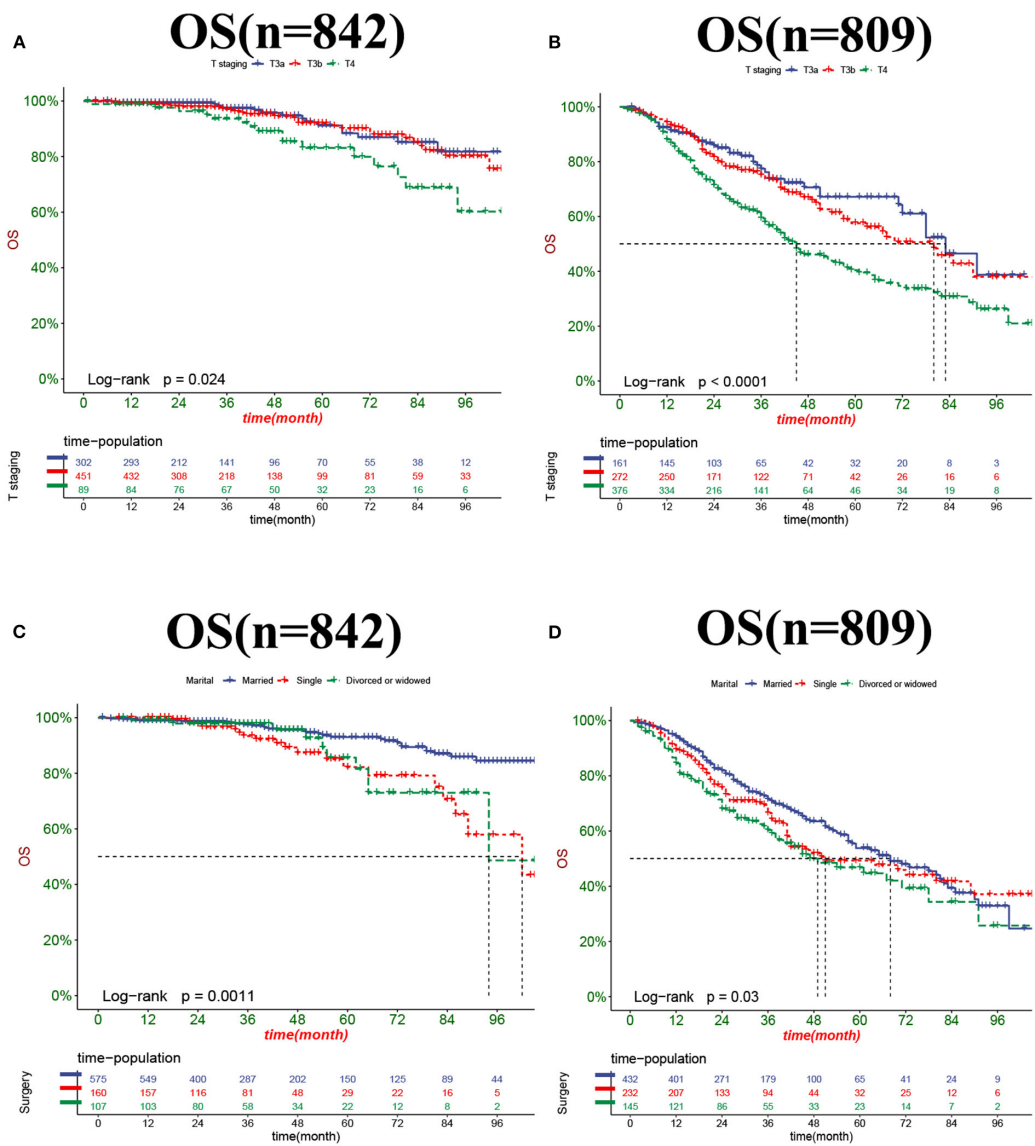
Kaplan-Meier curves for predicting OS in patients with different T stage **(A,B)** and marital status **(C,D)**.

**Figure 9:**
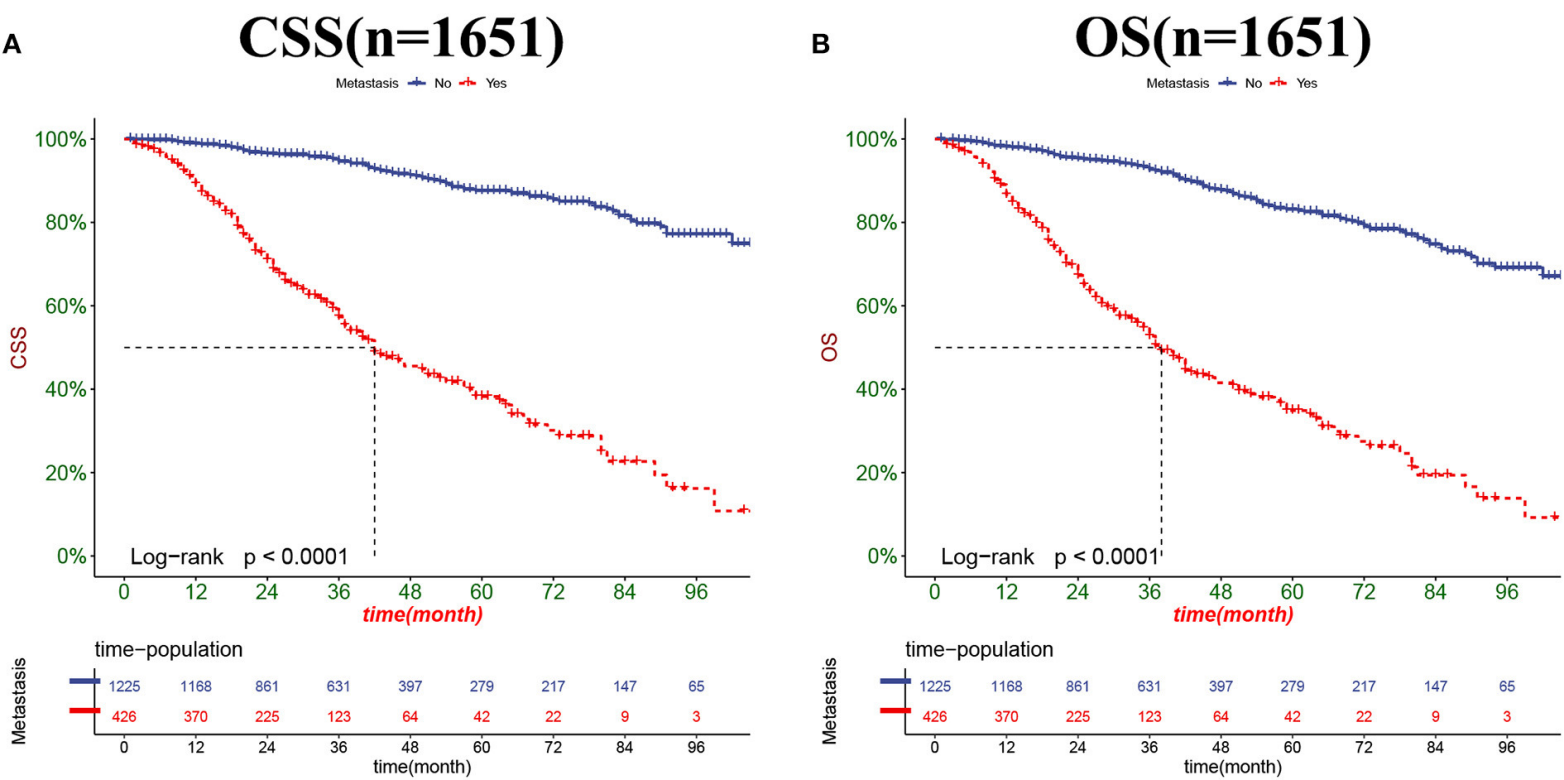
Kaplan-Meier curves for predicting CSS **(A)** and OS **(B)** in PC patients with distant metastases.

The DCA results showed that the nomograms for predicting CSS and OS had good clinical potential values in both training and validation groups (Figure 5). Furthermore, we calculated the optimal cutoff using the Youden index of ROC curve. Patients were divided into the high-risk group (total score ≥69.75) and the low-risk group (total score < 69.75) for the CSS analysis. In addition, 95.8 as a cut-off value divided patients into high-risk and low-risk groups for OS analysis. The K-M curve showed that the CSS and OS rates were significantly higher in low-risk group (Figure 6). In the low-risk group, The CSS rates at 3, 5, and 8 years were 98.1, 93.4, and 85.4%, respectively. In the low-risk group, the 3-, 5-, and 8-year OS rates were 96.8, 90.3, and 77.4%, respectively. We found that most patients underwent radical prostatectomy, followed by non-surgery. Patients undergoing radical prostatectomy have highest CSS and OS rates (Figures 7A,B). However, the higher the GS score, the lower the CSS and OS (Figures 7C,D). We analyzed differences in survival among patients with different T stages, the higher the T stage, the lower the OS (Figures 8A,B). The low-risk and high-risk groups showed that the married patients had the highest OS rate (Figures 8C,D). In addition, patients with distant metastases had lower CSS and OS than those without distant metastases (Figure 9).

## Discussion

As a senile disease, the onset of PC is closely related to age. The age of onset of PC is mostly concentrated in people over 65 years old (13). However, when PSA was first used for PC screening, it was not performed on patients of all ages, but primarily on patients over the age of 50. There are many studies on PC in the elderly patients, but few on PC in middle-aged patients of 50–65 years. Because middle-aged patients have fewer comorbidities, the prognosis is often better than older patients. However, high-risk PC patients often have a poor prognosis due to their vulnerability to metastatic recurrence. Therefore, the prognosis of middle-aged high-risk PC patients is worth our exploration. This study analyzed the risk factors in middle-aged high-risk PC patients and developed nomograms that could predict both OS and CSS.

High-risk PC was defined by the D'Amico risk stratification system as a patient with a T stage≥T2c, or PSA> 20 ng/mL, or GS≥8 (16). Thus, it shows that the high-risk patients have higher PSA, GS, and TNM stages. Clinically, in addition to traditional TNM staging, age, race, marital status, surgical method, radiotherapy and chemotherapy, PSA, and GS also affect the prognosis of patients. However, our study showed that for high-risk PC in middle age, independent risk factors affecting CSS mainly included GS, surgery and M stage, while independent risk factors affecting OS included GS, surgery, M stage, T stage, and marital status.

GS has been revised several times since it was proposed as an important influencing factor for evaluating the prognosis of patients with prostate cancer. The D'Amico risk stratification system is the most common risk stratification system for prostate cancer, divides the Gleason score into three groups (2–6, 7, and 8–10). Patients with the same GS score can have significant differences in prognosis. For example, studies have shown that patients with GS 4+3 scores have a worse prognosis than patients with 3+4 scores (17). Kryvenko et al. (18) proposed that patients with GS score 5+3, 4+4, 3+5 had significantly different. The prognosis of PC patients with different GS is more different, and a study shown that the prognosis of PC patients with GS 9-10 is worse than that of PC patients with GS 8, which is consistent with our results (19).

Surgery is one of the main treatment modalities for PC patients. At present, the main treatment methods for high-risk PC include radical prostatectomy plus long-term (2–3 years) androgen deprivation therapy (ADT) and radical prostatectomy plus pelvic lymph node dissection (PLND) (20, 21), indicating that radical prostatectomy is the main surgical method for high-risk PC. Our study also showed that it did confirm that the majority of middle-aged high-risk patients underwent radical prostatectomy. The K-M curve showed that middle-aged high-risk PC patients undergoing radical prostatectomy have the best prognosis. The study by Stephenson et al. (22) also showed that high-risk patients could benefit from radical prostatectomy, which is consistent with our conclusion. Middle-aged patients have fewer complications and better prognosis after radical prostatectomy. However, high-risk patients have a high risk of metastasis, and simple local tumor resection has no obvious benefit to patient survival.

High-risk patients have high mortality of due to risks such as recurrence and metastasis (23). The M stage refers to the distant metastasis of the tumor. At the same time, almost all cases of PC death have previously had metastasis, especially bone metastasis (24). Miyoshi et al. (25) developed a nomogram to predict overall survival in Japanese patients with metastatic prostate cancer in the bone. A novel nomogram to predict survival in prostate cancer was constructed by Liu et al. (26). The current nomograms of prostate cancer prognosis mostly include the traditional TNM stage (17, 27). These nomograms show that patients with higher T stage have a worse prognosis than those with distant metastases, which is consistent with our findings.

The relationship between marital status and oncological prognosis has been widely concerned. A trend-adjusted SEER database analysis by Chen et al. (28) confirms that marital status independently predicts NSCLC survival. The study by Ai et al. (29) showed that married patients with medullary thyroid carcinoma had better outcomes than unmarried patients. A population-based study showed that marital status was an independent predictor of laryngeal cancer survival, with widowed patients having lower survival (30). All the above studies have shown that marital status is an independent risk factor for the prognosis of cancer patients. A Meta-analysis by Guo et al. (31) on PC showed that unmarried status was associated with poorer mortality and survival outcomes in PC patients, especially in divorced and unmarried patients, which is consistent with our findings. This study showed that married patients had best outcomes for middle-aged high-risk PC patients. It may be because married patients receive more financial support and social attention. In addition, married patients may cooperate more actively and may receive more psychological comfort.

This study explored the risk factors affecting CSS and OS in middle-aged high-risk PC patients, and we constructed nomograms based on these risk factors. The internally validated model showed good accuracy that can assist clinicians and patients in decision-making. For example, in clinical practice, the highest CSS and OS rates are obtained with radical prostatectomy. In addition, if the 3-year survival rate is predicted to be low based on the nomogram, the patient can be guided for more intensive follow-up.

However, the research based on the SEER database itself has some limitations. First, for many PC patients, the main treatment modalities are active detection and ADT treatment, while the SEER database lacks data on active monitoring and ADT data. Secondly, the SEER data also lack information about patient BMI, smoking and drinking, and these factors may also be associated with the prognosis of PC patients. Thirdly, some patients were excluded because of incomplete information, so there may be some bias that is difficult to adjust for. Prospective studies are therefore necessary to confirm these findings. Finally, this study is a retrospective study and will be subject to certain bias. However, many key clinicopathological factors were still included and crossed internally so that the results were not subject to large error.

## Conclusions

We explored factors affecting CSS and OS in middle-aged high-risk PC patients, and we found that GS, surgery, M stage, marital status, and T stage were independent risk factors for OS. In addition, GS, surgery, and M stage were independent risk factors for CSS. We have developed nomograms to predict CSS and OS in middle-aged high-risk PC patients, and these models showed good accuracy and reliability through internal cross-validation, hoping to help clinicians and patients make better decisions.

## Data availability statement

Publicly available datasets were analyzed in this study. This data can be found at: https://seer.Cancer.gov/.

## Author contributions

GC, YL, and KH designed the study and reviewed and edited the article. XW, CZ, JW, and ZZ collected and analyzed the data. JW drafted the initial manuscript. GC, YL, KH, CZ, ZZ, and JW revised the article critically. All authors approved the final manuscript.

## Conflict of interest

The authors declare that the research was conducted in the absence of any commercial or financial relationships that could be construed as a potential conflict of interest.

## Publisher's note

All claims expressed in this article are solely those of the authors and do not necessarily represent those of their affiliated organizations, or those of the publisher, the editors and the reviewers. Any product that may be evaluated in this article, or claim that may be made by its manufacturer, is not guaranteed or endorsed by the publisher.

## References

[1] Nguyen-Nielsen M Borre M. Diagnostic and therapeutic strategies for prostate cancer. Semin Nucl Med. (2016) 46:484–90. 10.1053/j.semnuclmed.2016.07.002 27825428

[2] Siegel RL Miller KD Fuchs HE Jemal A. Cancer statistics, 2022. CA Cancer J Clin. (2022) 72:7–33. 10.3322/caac.21708 35020204

[3] Welch HG Albertsen PC. Prostate cancer diagnosis and treatment after the introduction of prostate-specific antigen screening: 1986-2005. J Natl Cancer Inst. (2009) 101:1325–9. 10.1093/jnci/djp278 19720969 PMC2758309

[4] Li J German R King J Joseph D Thompson T Wu XC. Recent trends in prostate cancer testing and incidence among men under age of 50. Cancer Epidemiol. (2012) 36:122–7. 10.1016/j.canep.2011.10.014 22112545

[5] Mottet N Bellmunt J Bolla M Briers E Cumberbatch MG De Santis M. EAU-ESTRO-SIOG guidelines on prostate cancer. Part 1: screening, diagnosis, and local treatment with curative intent. Eur Urol. (2017) 71:618–29. 10.1016/j.eururo.2016.08.003 27568654

[6] Cooperberg MR Broering JM Carroll PR. Time trends and local variation in primary treatment of localized prostate cancer. J Clin Oncol. (2010) 28:1117–23. 10.1200/JCO.2009.26.0133 20124165 PMC2834465

[7] Chang AJ Autio KA Roach M Scher HI. High-risk prostate cancer-classification and therapy. Nat Rev Clin Oncol. (2014) 11:308–23. 10.1038/nrclinonc.2014.68 24840073 PMC4508854

[8] D'Amico AV Whittington R Malkowicz SB Weinstein M Tomaszewski JE Schultz D. Predicting prostate specific antigen outcome preoperatively in the prostate specific antigen era. J Urol. (2001) 166:2185–8. 10.1016/S0022-5347(05)65531-0 11696732

[9] Liu X Wu Z Lin E Li W Chen Y Sun X. Systemic prognostic score and nomogram based on inflammatory, nutritional and tumor markers predict cancer-specific survival in stage II-III gastric cancer patients with adjuvant chemotherapy. Clin Nutr. (2019) 38:1853–60. 10.1016/j.clnu.2018.07.015 30075998

[10] Jiang S Zhao R Li Y Han X Liu Z Ge W. Prognosis and nomogram for predicting postoperative survival of duodenal adenocarcinoma: a retrospective study in China and the SEER database. Sci Rep. (2018) 8:7940. 10.1038/s41598-018-26145-6 29786691 PMC5962558

[11] Zuo Z Zhang G Song P Yang J Li S Zhong Z. Survival nomogram for stage IB non-small-cell lung cancer patients, based on the SEER database and an external validation cohort. Ann Surg Oncol. (2021) 28:3941–50. 10.1245/s10434-020-09362-0 33249521

[12] Tang X Zhou X Li Y Tian X Wang Y Huang M. A novel nomogram and risk classification system predicting the cancer-specific survival of patients with initially diagnosed metastatic esophageal cancer: a SEER-based study. Ann Surg Oncol. (2019) 26:321–8. 10.1245/s10434-018-6929-0 30357578

[13] Siegel DA O'Neil ME Richards TB Dowling NF Weir HK. Prostate cancer incidence and survival, by stage and race/ethnicity - United States, 2001-2017. Morb Mortal Wkly Rep. (2020) 69:1473–80. 10.15585/mmwr.mm6941a1 33056955 PMC7561091

[14] Swyer GI. Post-natal growth changes in the human prostate. J Anat. (1944) 78:130–45. 17104953 PMC1272501

[15] Zheng Y Lin SX Wu S Dahl DM Blute ML Zhong WD. Clinicopathological characteristics of localized prostate cancer in younger men aged < /= 50 years treated with radical prostatectomy in the PSA era: a systematic review and meta-analysis. Cancer Med. (2020) 9:6473–84. 10.1002/cam4.3320 32697048 PMC7520296

[16] D'Amico AV Whittington R Malkowicz SB Schultz D Blank K Broderick GA. Biochemical outcome after radical prostatectomy, external beam radiation therapy, or interstitial radiation therapy for clinically localized prostate cancer. J Am Med Assoc. (1998) 280:969–74. 10.1001/jama.280.11.969 9749478

[17] Zhu X Gou X Zhou M. Nomograms predict survival advantages of Gleason score 3+4 over 4+3 for prostate cancer: a SEER-based study. Front Oncol. (2019) 9:646. 10.3389/fonc.2019.00646 31380282 PMC6646708

[18] Kryvenko ON Williamson SR Schwartz LE Epstein JI. Gleason score 5+3=8 (grade group 4) prostate cancer-a rare occurrence with contemporary grading. Hum Pathol. (2020) 97:40–51. 10.1016/j.humpath.2019.11.002 31923450

[19] Safdieh JJ Schwartz D Weiner JP Nwokedi E Schreiber D. The need for more aggressive therapy for men with Gleason 9-10 disease compared to Gleason < /=8 high-risk prostate cancer. Tumori. (2016) 102:168–73. 10.5301/tj.5000475 26917408

[20] NCCN. Prostate Cancer Guidelines. (2015). Available online at: https://www.nccn.org/store/login/login.aspx?ReturnURL=http://www.nccn.org/professionals/physician.gls/pdf/prostate.pdf (accessed April 22, 2022).

[21] EAU. Guidelines on Prostate Cancer. (2015). Available online at: https://uroweb.org/guidelines/prostate-cancer (accessed April 24, 2022).

[22] Stephenson AJ Kattan MW Eastham JA Bianco FJ Jr Yossepowitch O Vickers AJ. Prostate cancer-specific mortality after radical prostatectomy for patients treated in the prostate-specific antigen era. J Clin Oncol. (2009) 27:4300–5. 10.1200/JCO.2008.18.2501 19636023 PMC3651598

[23] Payne HA Hughes S. Radical radiotherapy for high-risk prostate cancer in older men. Oncologist. (2012) 17(Suppl.1):9–15. 10.1634/theoncologist.2012-S1-09 23015680 PMC3593777

[24] Peisch SF Van Blarigan EL Chan JM Stampfer MJ Kenfield SA. Prostate cancer progression and mortality: a review of diet and lifestyle factors. World J Urol. (2017) 35:867–74. 10.1007/s00345-016-1914-3 27518576 PMC5472048

[25] Miyoshi Y Noguchi K Yanagisawa M Taguri M Morita S Ikeda I. Nomogram for overall survival of Japanese patients with bone-metastatic prostate cancer. BMC Cancer. (2015) 15:338. 10.1186/s12885-015-1330-x 25929438 PMC4423138

[26] Liu Y Li L Jiang D Yang M Gao X Lv K. A novel nomogram for survival prediction of patients with spinal metastasis from prostate cancer. Spine. (2021) 46:E364–73. 10.1097/BRS.0000000000003888 33620180

[27] Liu D Kuai Y Zhu R Zhou C Tao Y Han W. Prognosis of prostate cancer and bone metastasis pattern of patients: a SEER-based study and a local hospital based study from China. Sci Rep. (2020) 10:9104. 10.1038/s41598-020-64073-6 32499554 PMC7272631

[28] Chen Z Yin K Zheng D Gu J Luo J Wang S. Marital status independently predicts non-small cell lung cancer survival: a propensity-adjusted SEER database analysis. J Cancer Res Clin Oncol. (2020) 146:67–74. 10.1007/s00432-019-03084-x 31786738 PMC11804430

[29] Ai L Li N Tan HL Wei B Zhao YX Chen P. Effects of marital status on survival of medullary thyroid cancer stratified by age. Cancer Med. (2021) 10:8829–37. 10.1002/cam4.4388 34723436 PMC8683521

[30] Ding Z Yu D Li H Ding Y. Effects of marital status on overall and cancer-specific survival in laryngeal cancer patients: a population-based study. Sci Rep. (2021) 11:723. 10.1038/s41598-020-80698-z 33436991 PMC7803965

[31] Guo Z Gu C Li S Gan S Li Y Xiang S. Association between marital status and prognosis in patients with prostate cancer: a meta-analysis of observational studies. Urol J. (2020) 18:371–9. 10.22037/uj.v16i7.6197 33236334

